# Possible effects of a course in cardiovascular nursing on prehospital care of patients experiencing suspected acute coronary syndrome: a cluster randomised controlled trial

**DOI:** 10.1186/s12912-016-0175-1

**Published:** 2016-09-02

**Authors:** Birgitta Wireklint Sundström, Mats Holmberg, Johan Herlitz, Thomas Karlsson, Henrik Andersson

**Affiliations:** 1 PreHospen − Centre for Prehospital Research, University of Borås, Borås, Sweden; 2 Faculty of Caring Science, Work Life and Social Welfare, University of Borås, Borås, Sweden; 3 Department of Public Health and Community Medicine, Sahlgrenska Academy, Gothenburg, Sweden; 4 School of Health Sciences, University of Borås, SE-501 90 Borås, Sweden

**Keywords:** Acute coronary syndrome, Pain treatment, Lifeworld, Educational intervention, Ambulance services, Ambulance nurse

## Abstract

**Background:**

Current research suggests that nurses can influence the outcome for patients with acute coronary syndrome (ACS). The aim of this study has been to evaluate whether a course in cardiovascular nursing (CVN) can improve ambulance nurses’ (ANs’) prehospital care of patients experiencing suspected ACS, related to pain intensity.

**Methods:**

This is a cluster randomised controlled trial that was conducted in the ambulance services. Patients were allocated to one of two groups: in the first group, patients were treated by ANs who had attended the CVN course and in the second group patients were treated by ANs without this qualification. Inclusion criteria were: 1/pain raising suspicion of ACS, and 2/pain score ≥4 on a visual analogue scale (VAS). The primary outcome was the estimated intensity of pain or discomfort according to VAS 15 min after randomisation. Secondary outcomes were estimated intensity of pain or discomfort on admission to hospital and further requirement of pain treatment, as well as symptoms such as paleness and/or cold sweat; nausea and/or vomiting; anxiety, dyspnea, degree of alertness, respiratory depression and aggressiveness. A further secondary outcome measured was survival to 30 days. Lastly, a final diagnosis was made. A total of 38 ANs attended the CVN course. There were 1,747 patients who fulfilled the inclusion criteria.

**Results:**

The pain score did not differ significantly between the two groups fifteen minutes after randomisation (median value of VAS was 4.0 in both groups). On admission to hospital the pain score was significantly lower for patients treated by an AN who had attended the CVN course (*n* = 332) compared with those treated by an AN who had not attended the course (*n* = 1,415) (median 2.5 and 3.0 respectively, *p* = 0.001). The ANs who had attended the course used higher doses of morphine.

**Conclusions:**

An educational intervention with a CVN course did not relate significantly to more efficient pain relief in suspected ACS during the first 15 min. However, this intervention was associated with more effective pain relief later on in the prehospital setting. Thus, a CVN course for ANs appears to be associated with reduced pain intensity among patients experiencing suspected ACS. This result needs however to be confirmed in further trials.

**Trial registration:**

The ClinicalTrials.gov Protocol Registration System (registration number NCT00792181).

## Background

Acute coronary syndrome (ACS) is a common term used to describe a group of conditions resulting from acute myocardial ischemia (i.e. insufficient blood flow to heart muscle) and ranging from unstable angina pectoris to myocardial infarction [1].

Acute myocardial infarction may present less typical symptoms [2]. Nevertheless, pain or discomfort in the chest is one of the most common symptoms among patients calling the Emergency Medical Services (EMS) [3, 4]. Treatment of pain while caring for patients experiencing myocardial infarction is of paramount importance, not only for humane reasons but because the pain is associated with activation of the sympathetic nervous system that leads to vasoconstriction and intensifies the work of the heart.

The influence of nursing care on chest pain intensity in patients experiencing suspected ACS is an unexplored area in the ambulance services, especially research focusing on the ambulance nurses’ (ANs’) assessment and treatment of pain. There is a gap in knowledge regarding the assessment of pain, as well as of anxiety. These are aspects of the illness that are as important as the myocardial damage. The intensity of pain in ACS is a major healthcare interest since coronary heart disease is one of the most common causes of death worldwide [5]. There is a growing epidemic of coronary heart disease in low- and middle-income countries [6]. In many countries, cardiovascular disease causes more than twice as many deaths as cancer [7].

Nurses strive to provide direct contact with and care for the patient, and to relieve patients’ stress and anxiety through assessment, diagnosis and counselling. The ANs describe their care as focusing on the patient’s physical disorder while simultaneously taking her/his lifeworld into account [8]. This care approach meets the criterion of encountering patients with openness and flexibility, based on the patients’ own experiences [9]. According to current understanding of patients with acute myocardial infarction, effective communication is a key aspect of the clinician-patient relationship in prehospital care [10]. The relationship with the AN gives patients the feeling of being in a caring presence with gradually increasing comfort and hope of survival [11].

In the emergency department, the role of the registered nurse (RN) is assumed to be central to the provision of nursing care for patients with myocardial infarction [12]. RNs’ communication skills are vital to providing adequate pain and anxiety management. The role of nurses in a chest pain unit is reported to be vital when it comes to contributing to the relief of patients experiencing chest pain [13]. A similarly positive impact for patients with ACS has been shown in the coronary care unit regarding nurse-led early triage of chest pain patients [14].

However, knowledge is insufficient concerning ANs’ importance in the prehospital setting for optimal pain treatment in cases of ACS. The ambulance services should be considered not only as a mode of transport but also as a place for initial diagnosis, triage and treatment [15].

This study aims to evaluate whether a course in CVN improves ANs’ prehospital care of patients experiencing suspected ACS, related to pain intensity. According to this aim, the following hypothesis was formulated: “ANs who had attended a CVN course treated patients differently from those who had not attended a CVN course, regarding pain relief for patients experiencing suspected ACS.”

## Methods

### Research design and setting

A cluster randomised, controlled trial (RCT) was used involving an educational intervention for ANs. The patients included in the study were divided into two groups: A/those treated by an AN who had attended the CVN course (=educational intervention) and B/those who were treated by an AN without this qualification (=standard education), i.e. a control group.

In the EMS and the prehospital setting the procedure worked out in the following way: when the ambulance came to the patient with suspected ACS the ambulance staff included an AN with or without the CVN qualification. This meant in practice that if the AN had the CVN qualification, the patients belonged to the intervention group, while if the AN did not have the CVN qualification, they belonged to the control group. Thus the ANs’ qualification determined to which cluster the patient was allocated. Regardless of group allocation, and with the patients’ written informed consent, they were then randomised by an envelope method to treatment with midazolam or not. The primary results regarding midazolam were reported together with a consort flow diagram in Wireklint Sundström et al. [16].

The study setting was the EMS system in Western Sweden and in the city of Halmstad (six ambulance services and 1.5 million inhabitants).

### The characteristics of participants

Patients were included in the study if they complained of pain or discomfort that aroused suspicion of ACS and if they reported pain ≥4 on a visual analogue scale (VAS) [17]. That the requirement for reported pain stipulated a level of at least 4 was because of a decision to exclude patients with less severe pain in order to focus on patients who were expected with certainty to require pain relief treatment.

The exclusion criteria were: 1/Systolic blood pressure <100 mmHg, 2/age <18 years, 3/under the influence of alcohol, 4/under the influence of drugs, 5/benzodiazepine abuse, 6/dementia, disorientation, 7/communication problems, 8/symptoms judged as being caused by trauma and 9/secondary transports (i.e. treatment already started).

This study used patients’ outcomes to measure the effect of the CVN course for ANs. The entrance criteria for ANs were an RN qualification and a Bachelor of Science Degree. In Sweden there are two levels of AN working in the EMS: 1) RNs (Bachelor of Science Degree) and 2) RNs in Specialist Nursing, Prehospital Emergency Care (specialist course, i.e. one-year Master of Science Degree) [18]. Both groups (intervention and control) included ANs with the two possible education levels, but nobody had ever undergone the CVN course before. No prior power analysis was performed.

The study involved about 500 ANs, 60 ambulances and one ambulance boat. All the ambulances were staffed with at least one AN. The CVN courses ran from August 2007 to December 2009 (Table 1). Three courses took place, attended by a total of 38 ANs. The RCT started on 1 May 2008. The last patient was recruited on 31 December 2010. All in all, 1,747 patients took part in the study. Written informed consent was obtained from each patient.

**Table 1:** Timeline for the CVN courses, number av ANs attending and the RCT at overall six ambulance services

Year	Month	The CVN cours	The RCT
2007	August–December	The first CVN course ran: 15 ANs	
2008	May		The RCT started at four ambulance services
	August–December	The second CVN course ran: 11 ANs	
2009	April		The RCT started at two more ambulance services
	August–December	The third CVN course ran: 12 ANs	
2010	31 December		The RCT closed

### Educational intervention

The CVN course for ANs, amounting to 7.5 credits, II level (i.e. one-year Master’s Degree) (Table 2) was developed uniquely for this research project, and can therefore be regarded as a specialised education tailored to the needs of prehospital care. The CVN was a course including caring assessment and treatment, as well as clinical cardiology focusing on patients with ACS. This education was expected to deepen the ANs’ knowledge and increase their possibilities of relieving and treating chest pain and anxiety as well as providing cardiovascular treatment. A lifeworld approach (which refers to Edmund Husserl’s philosophy) [2] allowed caring and medicine to be applied simultaneously in a natural way [19]. The teaching strategies were based on the expectation that the patients would experience the inhibition of the anxiety accompanying their symptoms of pain and discomfort.

**Table 2:** Curriculum in Cardiovascular nursing (CVN) for ambulance nurses, 7.5 credits, II level (i.e. one-year Master’s Degree)

Objectives	After completing the course, the student should be able to
Knowledge and understanding	• Give an account of prehospital assessment and treatment of “chest pain/heart attack” with a focus on anxiety and pain, • Analyse and evaluate nursing needs and medical treatment and • Describe the meaning of a caring encounter with the patients.
Skills and abilities	• Analyse complex issues, formulate assessments and have preparedness in ambulance services relating to • Medical treatment for anxiety and pain relief of patients seeking for “chest pain/heart attack”, • Caring encounters and • Priority to the appropriate level of care.
Assessment and approach	• Reflect on the importance of an ambulance nurse’s attitude in a caring encounter with concurrent medical treatment, • Identify and analyse the existential issues, • Identify and analyse issues on the basis of gender and age differences and • iIdentify and analyse ethical dilemmas.

Various didactic methods were applied: lectures, seminars, workshops, drama, simulation training, and clinical practice at a Coronary Care Unit. Two theoretical examinations were included as well, culminating in the seminar “Prehospital care for patients experiencing ACS” (Table 3). The students then had mandatory articles to read as well as preparing a written survey based on an ambulance assignment experienced by the student. To gain a pass, students were required to follow the examination instructions when writing their survey; to base their survey on theory with clearly stated references; and to participate orally in the seminar.

**Table 3:** The seminar “Prehospital care for patients experiencing ACS”

Purpose	Assignment	Assessment/Grade	Literature
	At least one medical science and one caring science article should be used from the literature list. Free choice of other articles.		
To obtain improved knowledge of the assessment, treatment and relief of pain/discomfort and anxiety in patients calling the ambulance services for “chest pain/heart attack”.	Select an ambulance assignment that you have experienced involving the issues “chest pain/heart attack” and enter the scientific references to given care and treatment, i.e. explain the scientific support available today. - Describe the entire ambulance assignment – assessment, care and treatment – from the reception of information from the dispatch centre to the handover process. Note the patient’s estimated pain intensity (pain score). - What are the opportunities for and obstacles to relieving the patient’s experience in the prehospital care setting? - Discuss the caring and medical treatment (including pain and anxiety) in relation to the articles selected. What does the literature tell you? What do you not have scientific support for? What are the proven experiences? - Include gender and age differences in the analysis.	In order to gain the grade Approved, the student is required to have participated actively in the seminar and to have exhibited samples of reflected knowledge.	Bång et al., 2008. Lower mortality after prehospital recognition and treatment followed by fast tracking to coronary care compared with admittance via emergency department in patients with ST-elevation myocardial infarction. Int. J. Cardiol. 129 (3), 325–332. Frazier et al., 2002. Management of anxiety after acute myocardial infarction. Heart Lung 31 (6), 411–420. Huffman J. C., Stern T. A., 2003. The Use Of Benzodiazepines In The Treatment of Chest Pain: A Review of The Litterature. J. Emerg. Med. 25, 427–437. Lord B., Parsell B., 2003. Measurement of pain in the pehospital setting using a visual analogue scale. Prehosp. Disas. Med. 18 (4), 353–358. Moser et al., 2007. Impact of anxiety and perceived control on in-hospital complications after acute myocardial infarction. Psychosom. Med. 69, 10–16. Sandman, L., Nordmark, A., 2006. Ethical conflict in pre-hospital emergency care. Nurs. Ethics 13 (6), 592*–*607*.*

### Instruments

VASs are used to measure subjectives, such as pain [20]. The VAS instrument is a 100 mm scale on a straight line, of which the ends are the extreme limits of the sensation or feeling being measured. In this study the patients were asked to indicate the intensity of the pain, between 0 and 10, where 10 was the worst experience of pain. VAS was used for measuring the primary outcome and the first of the secondary outcomes.

The overall Cronbach’s coefficient alpha for the test-retest reliability on the three separate measures of pain intensity was 0.77 (i.e. acceptable internal consistency).

### Outcome measurements

All the measurements took place at three points in time: prior to randomisation, 15 min thereafter and on admission to a hospital. Evaluation of signs of heart failure and heart rhythm was carried out once, prior to randomisation. There was no written information apart from the regular guidelines for the personnel in ambulance services on how to assess outcomes.

#### Primary outcome measurement

The primary outcome was the estimated intensity of pain or discomfort according to VAS as perceived and assessed by the patients 15 min after randomisation. The nurses assisted the patients in handling VAS and wrote down the measurements.

#### Secondary outcome measurements

A number of secondary outcome measurements were also implemented. These included estimated intensity of pain or discomfort according to VAS as perceived and assessed by the patients on admission to hospital, as well as any further requirements for pain treatment. Symptoms were also recorded, such as paleness, and/or cold sweat, nausea and/or vomiting, anxiety, dyspnea, degree of alertness, respiratory depression, and aggressiveness. Furthermore, survival to 30 days and a final diagnosis of acute myocardial infarction and ACS were also recorded.

Acute myocardial infarction was defined in accordance with the diagnosis given in the hospital records. ACS was defined as a final hospital diagnosis of either myocardial infarction or unstable angina pectoris.

### Data analysis

Fisher’s exact test was used to test for differences in dichotomous variables and the Mann-Whitney *U* test was used for ordered/continuous variables. For survival analysis, Kaplan-Meier estimates were used and groups were compared using the log rank test. All tests are two-sided and a *p*-value of <0.05 for the primary outcome and <0.01 for other comparisons were considered statistically significant. To limit the risk of type I error due to multiple testing, we decided to use a nominal *p*-value of below 0.05 as significance level only for the primary outcome. All analyses were performed using SAS for Windows version 9.2. The power calculation for the trial was based on the estimated pain intensity as assessed by the patients and has been described in Wireklint Sundström (16).

## Results

Patients were divided into two groups: those who were treated by ANs with the CVN course (educational intervention) and those who were treated by ANs without the CVN course. All in all, 1,747 patients took part in the study. Among them 332 (18 %) were treated by an AN who had taken part in the educational intervention. There were no statistically significant differences between the patients in the two groups regarding age, sex and previous history (Table 4).

**Table 4:** Demographic characteristics of patients in each group

	Treated by AN with the CVN course		
	Yes	No	
	(*n* = 332)	(*n* = 1,415)	*p*
Age			
Years mean ± SD	69.1 ± 14.1	69.9 ± 13.3	0.38
Median (25^th^, 75^th^ percentile)	71 (59,80)	72 (61,81)	
Women (%)	45	44	0.85
Previous history (%)			
Myocardial infarction (12/47)^a^	37	37	0.95
Angina pectoris (10/62)	26	30	0.25
Hypertension (9/51)	41	43	0.71
Diabetes mellitus (8/42)	19	20	0.70
Heart failure (10/68)	13	15	0.43
Stroke (8/43)	8	10	0.25
Peripheral artery disease (12/47)	5	3	0.06
Chronic obstructive pulmonary disease (8/45)	8	9	0.51
Renal disease (8/45)	7	7	0.71
Cancer (10/41)	9	10	0.61
Smoking (%) (70/337)	15	20	0.08

^a^Number of patients with missing information in the two groups, respectively

### Symptoms and cardiopulmonary findings at randomisation

The ANs who had attended the CVN course reported more frequently that patients had anxiety at randomisation as compared with the other group. Otherwise, there was no significant difference between the two groups (Table 5).

**Table 5:** Symptoms and cardiopulmonary findings at randomisation

	Treated by AN with the CVN course		
	Yes	No	
	(*n* = 332)	(*n* = 1,415)	*p*
Symptoms (%)			
Pale, cold sweat (1/9)^a^	49	44	0.07
Nausea, vomiting (7/35)	25	27	0.62
Anxiety (9/30)	72	63	0.003
Dyspnea (6/33)	40	37	0.45
Localisation of pain (%)			
Chest (1/7)	98	97	0.08
Arm(s) (9/27)	44	46	0.54
Back (8/39)	20	25	0.07
Neck/jaw (9/46)	21	25	0.11
Stomach (6/45)	11	11	0.84
Estimated severity prior to randomisation (14/109)			
mean ± SD	6.4 ± 1.7	6.2 ± 1.6	0.16
median (25^th^, 75^th^ percentile)	6 (5,7.75)	6 (5,7.25)	
Cardiopulmonary findings			
Heart rate: beats/min mean ± SD	87.9 ± 25.1	86.2 ± 24.6	0.21
(1/13) median (25^th^, 75^th^ percentile)	83 (70,100)	82 (70,98)	
Systolic blood pressure: mmHg mean ± SD	150.1 ± 26.8	152.2 ± 27.5	0.18
(3/15) median (25^th^, 75^th^ percentile)	150 (130,169)	150 (132,170)	
Oxygen saturation: % mean ± SD	95.6 ± 3.9	95.9 ± 3.5	0.35
(6/38) median (25^th^, 75^th^ percentile)	97 (95,98)	97 (95,98)	
Respiratory rate: breath/min mean ± SD	18.3 ± 5.3	18.4 ± 5.1	0.29
(26/135) median (25^th^, 75^th^ percentile)	16 (15,20)	18 (15,20)	
Signs of heart failure (ascultatory rales) (%):	3	5	0.36
(125/695)			
Sinus rhythm (%): (8/63)	80	80	0.88

^a^Number of patients with missing information in the two groups, respectively

### Treatment prior to hospitalisation

Among patients receiving nitrates, those treated by an AN who had attended the CVN course received lower doses compared to those treated by an AN who had not, but on the other hand the amount of morphine was higher in the former group (educational intervention). Otherwise no statistically significant difference was found (Table 6).

**Table 6:** Treatment prior to hospitalisation

	Treated by AN with the CVN course		
	Yes	No	
	(*n* = 332)	(*n* = 1,415)	*p*
Oxygen (%) (1/2)^a^	77	82	0.051
Mean (median) dose^b^; l/min	3.9 (3)	3.7 (3)	0.11
Aspirin (%) (2/3)	62	66	0.20
Mean (median) dose^b^; mg	298 (320)	294 (320)	0.26
Clopidogrel (%) (1/4)	10	9	0.52
Mean (median) dose^b^; mg	565 (600)	571 (600)	0.62
Nitrates (%) (2/2)	80	85	0.02
Mean (median) dose^b^; mg	0.74 (0.8)	0.78 (0.8)	0.004
Betablockers (%) (2/5)	5	4	0.36
Mean (median) dose^b^; mg	8.8 (5)	11.3 (15)	0.05
Midazolam (%) (1/1)	46	50	0.25
Mean (median) dose^b^; mg	1.13 (1.00)	1.12 (1.00)	0.91
Morphine (%) (2/4)	82	82	0.81
Mean (median) dose^b^; mg	6.4 (5.0)	5.5 (5.0)	<0.0001

^a^Number of patients with missing information in the two groups, respectively

^b^Of those patients receiving treatment

### ECG findings

Apart from pathological Q-waves being more frequent in patients treated by an AN who had attended the CVN course, no significant difference in terms of ECG recorded, ECG sent or ECG findings between the two groups was observed.

### Symptoms after randomisation prior to hospital admission

#### Localisation of pain or discomfort

No statistically significant differences were observed at randomisation or 15 min thereafter. However, a significantly lower proportion of patients treated by an AN who had attended the CVN course had back pain on arrival in hospital (Table 7).

**Table 7:** Symptoms after randomisation prior to hospital admission

	Treated by AN with the CVN course		
	Yes	No	
	(*n* = 332)	(*n* = 1,415)	*p*
Localisation of pain or discomfort (%)			
15 min after randomisation			
Chest (15/73)^a^	78	84	0.03
Arm (s) (21/91)	24	31	0.02
Back (25/106)	14	19	0.06
Neck/jaw (22/110)	16	19	0.25
Stomach (20/110)	8	8	0.91
On admission to hospital			
Chest (33/133)	60	67	0.03
Arm(s) (40/141)	16	22	0.05
Back (38/144)	7	15	0.0008
Neck/jaw (34/154)	9	14	0.02
Stomach (34/146)	7	7	1.00
Chest pain (VAS)			
15 min after randomisation			
Pain score mean ± SD	4.0 ± 2.2	4.2 ± 2.0	0.24
(28/146) median (25^th^, 75^th^ percentile)	4 (3,5.5)	4 (3,5.5)	
Pain score < 4 (%) (28/146)	24	20	0.16
Pain score = 0 (%) (28/146)	11	5	0.0002
Decrease in pain score^b^ (%) (29/152)	85	80	0.05
On admission to hospital			
Pain score mean ± SD	2.6 ± 2.1	3.0 ± 2.1	0.001
(29/167) median (25^th^, 75^th^ percentile)	2.5 (0.75,4)	3 (1.25,4.45)	
Pain score < 4 (%) (29/167)	53	47	0.05
Pain score = 0 (%) (29/167)	23	14	0.0001
Decrease in pain score^b^ (%) (30/173)	93	92	0.63
Other symptoms (%)			
15 min after randomisation			
Pale, cold sweat (10/77)	31	29	0.59
Nausea, vomiting (17/95)	14	15	0.60
Anxiety (21/100)	38	34	0.19
Dyspnea (19/98)	19	18	0.69
On admission to hospital			
Pale, cold sweat (29/124)	18	20	0.58
Nausea, vomiting (32/141)	10	11	0.92
Anxiety (42/155)	19	19	1.00
Dyspnea (36/150)	9	11	0.34
Alertness (%)			
15 min after randomisation (50/252)		0.06*	
Awake	93	90	
Dozy, confused	6	9	
Very dozy, confused	1	1	
Unconscious	0	<1	
On admission to hospital (51/253)		0.31*	
Awake	92	90	
Dozy, confused	8	8	
Very dozy, confused	<1	2	
Unconscious	0	<1	
Respiratory depression (%)			
15 min after randomisation (49/245)	1	<1	0.71
On admission to hospital (52/250)	<1	<1	1.00
Aggressiveness (%)			
15 min after randomisation (49/249)	<1	<1	0.48
On admission to hospital (52/252)	<1	<1	1.00

^a^Number of patients with missing information in the two groups, respectively

^b^From prior to randomisation

*p-value refers to the overall distribution of patients in the four groups

#### Chest pain

The primary outcome of intensity of pain 15 min after randomisation did not differ significantly between the two groups (*p* = 0.24). However, on admission to hospital the pain score was lower among patients treated by an AN who had attended the CVN course (*p* = 0.001). The proportion of patients free from pain (i.e. a pain score of 0) was significantly higher among patients treated by an AN who had attended the CVN course, both 15 min after randomisation and on admission to hospital.

#### Other symptoms, alertness, respiratory depression and aggressiveness

There was no significant difference between groups.

### Complications and final diagnosis

There was no statistically significant difference in complications either prior to or after arrival in hospital. Survival to 30 days did not differ significantly, nor did the proportion of patients with a final diagnosis of acute myocardial infarction.

## Discussion

Our primary hypothesis that treatment by an AN who had attended a CVN course would result in more effective pain relief 15 min after start of treatment in suspected ACS was not confirmed. However, there are a number of secondary outcome findings that suggest a connection between treatment allocation and outcome. These results should however be regarded as hypothesis-generating, since they all deal with secondary outcomes, and therefore need to be confirmed in further studies.

The results show that ANs who had attended the CVN course gave significantly higher doses of morphine (i.e. average 1 mg more morphine) compared to ANs without this qualification. This is important, especially since there were no significant differences in demographics, previous history or cardiopulmonary findings between the two groups. Despite this, the ANs who had attended the CVN course showed the courage to use their knowledge in pain treatment with morphine in line with earlier recommendations [21]. Thus, the CVN course, although not associated with pain relief during the first 15 min, might improve patients’ pain relief in a prolonged perspective. These findings are exclusive for the ambulance services and harmonise with three earlier studies from the ED [21–23]. With regard to intensive care nurses it is urgently necessary to reinforce their pain education [24]. We emphasise that national consultation is needed concerning guidelines preparing RNs for patient care when it comes to therapy for patients with ACS [25]. According to a European survey [26] there is variability in the content, teaching, learning and evaluation methods in post-registration cardiovascular nurse education programmes in Europe. The emphasis on interdisciplinary education that will foster more effective teamwork across multidisciplinary teams can offer many potential benefits. Pain relief should be approached using a holistic, systematic and evidence-based model [27].

In compliance with earlier studies we highlight the fact that healthcare providers can influence the outcome for patients with ACS by providing skilled, efficient, and coordinated prehospital care [28], and we also stress the need of both pain and anxiety relief in line with Frazier et al. [29].

We found that ANs who had completed the CVN course reported patients’ anxiety at the time of randomisation with significantly greater frequency. One reasonable connection to education and training in cardiovascular nursing might be that improved knowledge of nursing care for patients with suspected ACS has increased the RNs’ awareness of and ability to recognise symptoms and signs of ACS. Therefore these results are in line with earlier studies that have noticed the need for management of anxiety after acute myocardial infarction [29] and that vulnerability and feelings of anxiety constitute a well-known experience for patients experiencing an acute myocardial infarction [30].

However, the present results are not sufficiently satisfactory on all points. Only 23 % and 14 % respectively of the patients were free from pain on admission to hospital (shown in Table 7). These are remarkably poor results, especially when related to recommendations in the American Heart Association guidelines [31] for pain relief with the use of morphine in prehospital treatment. Inadequate analgesia [32, 33] is still an issue in the prehospital setting of acute chest pain.

Our findings suggest that more education and training in cardiovascular nursing are utterly essential in order to meet patients’ need for and right to pain treatment and pain relief when ACS is suspected by the ambulance services, and also in order to meet new demands on professional development for ANs. It is likely that this type of education needs to be reinforced and intensified even further.

### Strengths and limitations

The present study is to our knowledge unique with regard to the lifeworld and interdisciplinary approach towards ACS with its endeavour to develop an understanding of the possible connections between ANs having attended the CVN course and patients’ estimated intensity of pain in the prehospital setting. We argue that this approach is a strength.

One limitation might be that both groups of nurses consisted of both RNs with only a Bachelor of Science Degree and RNs with this degree and also with a specialist Master’s Degree. This means that theoretically, knowledge in Caring Science/Nursing varied in both groups. Another possibly more realistic limitation might be that before the course we did not measure the attitudes and opinions of the ANs involved concerning pain treatment with morphine, nor their overall interest in caring for these patients and patients experiencing from ACS. Perhaps there was a selection bias because the nurses who applied to the CVN course already had a positive attitude to pain treatment and caring for this patient group.

Finally, achieving a high rate of reliability must be regarded as problematical when there are many persons involved in the collection of data. Thus, involving several hundred RNs opens up the situation for deficiencies in implementation. The ambulance services are moreover a field that is inexperienced with regard to research. Taken all together, there are a number of circumstances that might lead to lack of reliability. We must take note of the fact that there is a risk of low reliability in this and other equivalent studies. The data in this study is also too limited to be able to function as a basis for a discussion about generalisability. These results should therefore solely be regarded as hypothesis-generating.

## Conclusions

An educational intervention was not shown to relate significantly to the primary outcome of patients scoring lower on the VAS pain scale after 15 min. Thus, patients with estimated intensity of pain ≥4 raising suspicion of ACS and treated by an AN who had recently completed the CVN course did not have less pain during the first 15 min after the ambulance’s arrival on scene. However, our data suggests that such an intervention was connected to more effective pain relief later on but still in the prehospital setting.

This study underlines the ANs’ potential for assisting patients to experience less pain and achieve relief of symptoms despite illness deriving from ACS. Therefore, the results should encourage increased investments in the education of ANs whose work brings them into close contact with these patients. Further studies should be carried out for additional confirmation of the results found in the present trial and to identify factors associated with the prehospital care provided.

## Abbreviations

ACS: Acute coronary syndrome
AN: Ambulance nurse
CVN: Cardiovascular nursing
ECG: Electro cardiogram
EMS: Emergency medical services
RCT: Randomised controlled trial
RN: Registered nurse
VAS: Visual analogue scale
